# Seeing Picasso: an investigation into the visual system of the triggerfish *Rhinecanthus aculeatus*

**DOI:** 10.1242/jeb.243907

**Published:** 2022-04-08

**Authors:** Karen L. Cheney, Jemma Hudson, Fanny de Busserolles, Martin Luehrmann, Abigail Shaughnessy, Cedric van den Berg, Naomi F. Green, N. Justin Marshall, Fabio Cortesi

**Affiliations:** 1 School of Biological Sciences, The University of Queensland, Brisbane, QLD 4072, Australia; 2 Queensland Brain Institute, The University of Queensland, Brisbane, QLD 4072, Australia

**Keywords:** Behaviour, Colour vision, Opsin, Gene expression, Visual pigment, Retinal topography, Visual acuity

## Abstract

Vision is used by animals to find food and mates, avoid predators, defend resources and navigate through complex habitats. Behavioural experiments are essential for understanding animals' perception but are often challenging and time-consuming; therefore, using species that can be trained easily for complex tasks is advantageous. Picasso triggerfish, *Rhinecanthus aculeatus*, have been used in many behavioural studies investigating vision and navigation. However, little is known about the molecular and anatomical basis of their visual system. We addressed this knowledge gap here and behaviourally tested achromatic and chromatic acuity. In terms of visual opsins, *R. aculeatus* possessed one rod opsin gene (*RH1*) and at least nine cone opsins: one violet-sensitive *SWS2B* gene, seven duplicates of the blue–green-sensitive *RH2* gene (*RH2A*, *RH2B*, *RH2C1-5*) and one red-sensitive *LWS* gene. However, only five cone opsins were expressed: *SWS2B* expression was consistent, while *RH2A*, *RH2C-1* and *RH2C-2* expression varied depending on whether fish were sampled from the field or aquaria. Levels of *LWS* expression were very low. Using fluorescence *in situ* hybridisation, we found *SWS2B* was expressed exclusively in single cones, whereas *RH2A* and *RH2Cs* were expressed in opposite double cone members. Anatomical resolution estimated from ganglion cell densities was 6.8 cycles per degree (cpd), which was significantly higher than values obtained from behavioural testing for black-and-white achromatic stimuli (3.9 cpd) and chromatic stimuli (1.7–1.8 cpd). These measures were twice as high as previously reported. This detailed information on their visual system will help inform future studies with this emerging focal species.

## Abstract (precis)

**Summary:** The triggerfish *Rhinecanthus aculeatus* is an ideal species for behavioural investigations of visual processes. They express six opsin proteins in different photoreceptors, and their visual acuity is significantly better for achromatic than for chromatic stimuli.

## INTRODUCTION

Behavioural evidence of colour vision in non-human animals was first demonstrated in bees over 100 years ago (von Frisch, 1914). Since then, behavioural experiments have been conducted with a range of animal species to investigate the mechanisms that underly colour and other visual processes (reviewed in Kelber et al., 2003). Furthermore, psychophysical experiments have explored higher-order neural processes such as colour constancy (e.g. Olsson et al., 2016), generalisation (e.g. Baddeley et al., 2001) and categorisation (e.g. Caves et al., 2018; Jones et al., 2001). However, behavioural experiments with animals are often challenging and time-consuming; therefore, it can be advantageous to use species that can be trained easily and perform complex tasks well. Historically, terrestrial animals such as honeybees, birds (e.g. chicks, finches, budgerigars, blue tits), lizards and mice have performed well in such studies (e.g. de Ibarra et al., 2002; Lind and Kelber, 2011; Silvasti et al., 2021; Stoddard et al., 2020). Focal teleost species have included zebrafish, goldfish, sticklebacks, cichlids, guppies and damselfish (e.g. Escobar-Camacho et al., 2019; Neumeyer, 1985, 1986, 1992; Risner et al., 2006; Sibeaux et al., 2019, 2008).

Over the past decade, a coral reef triggerfish, *Rhinecanthus aculeatus* (Linnaeus 1758) (commonly known as the Picasso or lagoon triggerfish), has been used in a number of studies on visual processes, including the role of double cones (two single cones joined together) (Pignatelli et al., 2010), colour discrimination thresholds (Champ et al., 2016; Cheney et al., 2019), the perception of visual illusions (Simpson et al., 2016), the impact of caustics on object detection (Matchette et al., 2020), the segregation of objects (Mitchell et al., 2017) and context-dependent luminance perception (van den Berg et al., 2020). *Rhinecanthus aculeatus* has also been used to understand how animals collect, process and use spatial information to accurately navigate through their environment (Karlsson et al., 2019 preprint), for bioinspired fish robots (Hu et al., 2006) and to provide an example of unusual sound production in teleosts (Parmentier et al., 2017). They are ideal for behavioural studies as they are relatively easy to keep, have a bold personality, and can be trained for complex tasks using operant conditioning. However, a detailed molecular and anatomical investigation of their visual system is needed to help interpret behavioural results, which we present in this study.

In common with other vertebrates, the photoreceptor cells of fish are in a backward-facing layer at the back of the retina, and contain visual pigments, constructed from an opsin protein with a centrally bound light-sensitive retinal chromophore (reviewed in Musilova et al., 2021). The visual pigment absorbs light and initiates the phototransduction cascade, which converts the light input into electric signals. These signals are carried through several neuronal layers before reaching the ganglion cells that converge into the optic nerve. It is the number and spacing of the ganglion cells that provide the upper limit for spatial resolution or visual acuity. Teleosts often show specialisation in ganglion- and photoreceptor-cell densities and distribution that reflect their specific ecologies. Fishes that live in highly complex 3D visual environments such as coral reefs often have concentric areas of higher cell densities that improve visual resolution along their main visual axes. In contrast, teleosts that occupy simpler visual environments, such as at the sand–water interface, have horizontal streaks of higher cell densities that assist in scanning the horizon for food or predators (reviewed in Cortesi et al., 2020).

To perceive colour, animals must have at least two photoreceptor types with different spectral sensitivities, and the relative excitation ratios from each photoreceptor type are compared in a colour opponency process. The peak spectral sensitivity (λ_max_) of the visual pigment depends both on variability in key opsin amino acids in the retinal binding pocket and the chromophore type used: vitamin A1-based pigment is shorter shifted compared with vitamin A2-based chromophore (reviewed in Carleton et al., 2020). Vertebrate visual opsins are classified into five types based on their photoreceptor specificity, phylogeny and the λ_max_ they confer. These five visual opsins were most likely already present in the vertebrate ancestor (Collin et al., 2003) and encode one rod opsin (rhodopsin, RH1, teleost λ_max_=447–525 nm) and four cone opsins: a short-wavelength-sensitive protein class 1 opsin (SWS1) maximally sensitive to UV–violet wavelengths (347–383 nm λ_max_), a second short-wavelength-sensitive class opsin (SWS2) maximally sensitive to the violet–blue part of the spectrum (397–482 nm λ_max_), a middle-wavelength-sensitive class 2 rhodopsin-like opsin (RH2) maximally sensitive to blue–green wavelengths (452–537 nm λ_max_) and a long-wavelength-sensitive class opsin (LWS) maximally sensitive to the green–red part of the light spectrum (501–573 nm λ_max_) (Carleton et al., 2020). SWS proteins are usually found in single cones, whereas RH2 and LWS opsins occur in double cones (Carleton et al., 2020).

Previously, the spectral sensitivities of *Rhinecanthus aculeatus* photoreceptors were measured using microspectrophotometry (MSP) (Cheney et al., 2013). *Rhinecanthus aculeatus* has a single cone that houses a short-wavelength pigment (413 nm λ_max_), whereas medium- (480 nm λ_max_) and long-wavelength pigments (528 nm λ_max_) are housed separately in the two members of the double cone. *Rhinecanthus aculeatus* also use the two members of the double cone independently in colour vision, facilitating trichromatic colour perception (Pignatelli et al., 2010). This suggests that – unlike birds, for example – some fish may not have photoreceptors specialised for either chromatic or achromatic vision (but see the review by Baden, 2021, on visual circuits in zebrafish). However, little is known about the molecular basis of vision in this species. Therefore, we first used whole genome sequencing and high-throughput RNA sequencing (RNAseq) to determine opsin gene repertoire and opsin expression. Second, with each expressed opsin sequence and known data on spectral tuning sites, the spectral sensitivity for each opsin class was predicted to examine whether it compared with previously published MSP data (Cheney et al., 2013). We then used fluorescence *in situ* hybridisation (FISH) to determine opsin specificity to single and double cone types.

Finally, we used an integrative approach to examine visual acuity in this species using retinal topography of photoreceptor and ganglion cells across the retina, and behavioural acuity experiments of both achromatic and isoluminant chromatic gratings using a paired-choice test with square wave gratings. Previous behavioural testing with this species using achromatic black-and-white gratings suggests that this species has a visual acuity of 1.75 cpd (Champ et al., 2014). However, anatomical investigations of triggerfish suggest higher possible visual acuity of 3.4 cpd for ganglion cells (Champ et al., 2014). Spatial contrast sensitivity may be higher for achromatic gratings than for chromatic gratings, which has been shown in other vertebrates, including humans and birds (Lind and Kelber, 2011; Mullen, 1985). In this study, we provide a deeper investigation into the visual system of this emerging focal species, which we hope will inform future studies of visual perception and navigation.

## MATERIALS AND METHODS

### Study species and specimen collection

*Rhinecanthus aculeatus* are common reef fish found on shallow sub-tidal reef flats across the Indo-Pacific region and are generalist omnivores known to feed on a varied diet including algae, detritus, molluscs and crustaceans (Randall et al., 1997). Individuals (*n*=23) were caught using clove oil and hand nets from shallow reefs surrounding Lizard Island, Great Barrier Reef, Australia, or obtained from an aquarium supplier (Cairns Marine Pty Ltd, Cairns, Australia) between 2017 and 2020. Fish ranged in size from 8.1 to 20.6 cm (standard length, SL) and were collected under a Queensland General Fisheries Permit (183990) and Great Barrier Reef Marine Park Authority Permit (G16/38497.1). Sex and age were not determined. Experiments were approved by the University of Queensland Animal Ethics Committee (2017/AEC000077 and QBI/304/16).

For molecular and anatomical techniques, fish (*n*=15) were anaesthetised with clove oil (10% clove oil; 40% ethanol; 50% seawater) and then euthanised. Eyes were enucleated, then the cornea and lens were removed. This species has a yellow corneal pigment (Siebeck and Marshall, 2001), the density of which increases across the cornea during the day (N.F.G., unpublished observations). Depending on the analysis, the eyes were preserved in different fixative solutions. Eyes that were allocated to retinal mapping were fixed overnight in 4% paraformaldehyde (PFA) and stored at 4°C. Following this, these eyes were stored in 0.1 mol l^−1^ phosphate-buffered saline (PBS; pH 7.4) until further analysis. For RNA sequencing, retinas were dissected out of the eyecup and preserved in RNAlater (Ambion) at −20°C until extraction. For FISH, retinas were prepared following the protocol in Barthel and Raymond (2000) and fixed in 4% PFA at 4°C overnight, washed twice for 5 min in PBS and rinsed briefly in 70% MetOH, before being transferred to 100% methanol and stored at −20°C until use. One individual was fin clipped and the tissue was preserved on 100% ethanol for genome sequencing.

For behavioural experiments, fish (*n*=8) were transported to the University of Queensland and housed in individual tanks (89×41×22 cm). Each tank had continuously flowing water supplied by a sump system and air stones to oxygenate the water.

### Transcriptome and genome sequencing, quality filtering and *de novo* assembly

The retinal transcriptomes of five *R. aculeatus* were sequenced according to Musilova et al. (2019). One individual was euthanised immediately after capture from the reefs off Lizard Island, Great Barrier Reef, and four individuals were euthanised after being in aquaria at the University of Queensland for between 10 and 18 months. In summary, total RNA was extracted using an RNeasy midi kit (Qiagen), and the quality and concentration of the RNA was checked using a Eukaryotic Total RNA Nanochip on an Agilent 2100 BioAnalyzer. The transcriptome sequencing and RNAseq libraries (paired end, 150 bp insert) were outsourced to Novogene (novogene.com). The transcriptome filtering and *de novo* assembly was performed following the protocol described in de Busserolles et al. (2017). Briefly, the raw reads were uploaded to the Genomics Virtual Laboratory (v.4.0.0) (Afgan et al., 2015) on the Bioinformatics platform Galaxy Australia (https://usegalaxy.org.au/). Using FastQC (Galaxy v.0.72), the quality of the sequences was assessed. They were then quality filtered using Trimmomatic (Galaxy v. 0.36.6) (Bolger et al., 2014), followed by the *de novo* assembly using Trinity (Galaxy v.2.8.5) (Haas et al., 2013). The newly sequenced samples were subsequently combined with three of our previously sequenced wild-caught samples from Lizard Island (Musilova et al., 2019) to make up our *R. aculeatus* opsin expression dataset.

To identify the visual opsin genes from the transcriptomes, further analyses were performed following the detailed protocol in de Busserolles et al. (2017). The assembled transcripts of *R. aculeatus* were mapped to the opsin gene sequences of the dusky dottyback (*Pseudochromis fuscus*; GenBank accession no. KP004335.1) using the medium sensitivity (30% maximum mismatch between transcripts) in Geneious v.2020.1.2 (www.geneious.com). *Pseudochromis fuscus* was chosen because of its relatively close phylogenetic relationship with *R. aculeatus*, as well as having representatives from all five visual opsin gene classes. To ascertain that all opsin genes were correctly assembled, and lowly expressed genes were picked up in the assembly, the filtered, unassembled transcriptome reads were then back-mapped against the extracted opsins' sequences using medium-low sensitivity (20% maximum mismatch between reads). For *RH2*, we detected extra copies that were only partially assembled or that were misassembled. Hence, in this case we used a read mapping approach to disentangle similar gene copies as per de Busserolles et al. (2017) and Musilova et al. (2019). Filtered, unassembled reads were mapped against the *P. fuscus RH2* reference using medium-low sensitivity settings (20% maximum mismatch between reads). We then extracted copy-specific reads by moving along the reference from single-nucleotide polymorphism (SNP) to SNP. Paired-end information allowed us to bridge the gaps between the SNPs. If the coding region was not completely recovered, we re-mapped unassembled reads using the consensus as a template with low sensitivity settings (0–2% maximum mismatch between reads). This allowed us to reconstruct the whole coding region of all expressed *RH2* copies.

To make sure that we did not miss any visual opsin genes that were not expressed in our samples, we sequenced a draft genome for *R. aculeatus* (ID Olaf) using Illumina short-read technology. Genomic DNA was extracted from a fin clip using the Qiagen DNeasy Blood & Tissue kit according to the manufacturer’s protocol (qiagen.com). DNA quality control (Agilent 5400, agilent.com), library preparation and sequencing (paired end 150 bp, 350 bp insert) was outsourced to Novogene (novogene.com). Visual opsin genes were subsequently extracted from the genomic raw reads (212,097,824 paired-end fragments, 31.8 Gb) using the read-mapping approach as outlined above and detailed in Musilova et al. (2019). The only difference was that for the genome, we used the raw reads and mapped them against the single exons of the *P. fuscus* opsins to avoid long-repetitive intronic sequences. The extracted opsin genes from the genome were then combined with the ones mined from the transcriptomes to generate an *R. aculeatus* visual opsin gene dataset.

 We confirmed opsin gene identity by using BLAST (https://blast.ncbi.nlm.nih.gov/) and by phylogenetic reconstruction. To obtain the opsin gene phylogeny, the opsin gene sequences of *R. aculeatus* were aligned with a reference dataset (obtained from GenBank, https://www.ncbi.nlm.nih.gov/genbank) using MAFFT v.7.450 (Katoh and Standley, 2013) with the L-INS-I settings. On the CIPRES platform, jModeltest v.2.1.6 was used to select the most appropriate model of sequence evolution. Following this, MrBayes v.3.2.7a (Ronquist et al., 2012) was used to infer the phylogenetic relationship between the genes. The following parameters were applied: GTR+I+γ model, with two independent MCMC searches (four chains each), 10 million generations per run, a tree sampling frequency of 1000 generations, and a burn-in of 25%.

### Opsin gene expression

For quantitative gene expression, the filtered reads were mapped to the coding regions of the identified opsin genes with high-specificity settings (98% identity, 80 bp minimum read overlap) as per the protocol in de Busserolles et al. (2017). Only one single cone gene, SWS2B, was expressed. For the other opsins, the gene-specific proportional expression was calculated as the fraction of all expressed for double cone genes *RH2*s and *LWS*, and all opsin genes for *RH1* (see Yourick et al., 2019, for a detailed discussion on opsin gene expression calculations) (Table S2).

For each opsin gene (*i*), the read count (*R_i_*) was normalised to the length of its coding sequence (*L_i_*; bp):
(1)

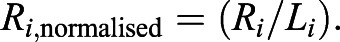


The proportion of rod opsin expressed was calculated as the proportion of *RH1* (*p_i_*_,rod_) relative to the total normalised opsin expression (*T*_opsin_):
(2)

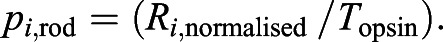


The proportional expression of double cones (*p_i_*_,DC_) was calculated as the proportion of the normalised opsin expression (*R_i_*_,normalised_) out of the total normalised expression for double cones (*T*_DC_):
(3)

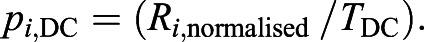


### Fluorescence *in situ* hybridisation (FISH)

Dual-labelling FISH was performed on whole-mount retinas of one adult, wild-caught *R. aculeatus* following standard protocols (Barthel and Raymond, 2000; Dalton et al., 2015). Previously extracted retinal mRNA was reverse transcribed using a High-Capacity RNA-to-cDNA Reverse Transcription Kit (Applied Biosystems). Riboprobe templates were synthesized from cDNA via standard PCR using Q5 High Fidelity DNA polymerase (New England Biolabs) and opsin-specific primers (Table S1). Amplicons were isolated via gel-electrophoresis and gel extraction (Qiagen Gel Extraction Kit), followed by enrichment PCR using gel-extracted amplicons as cDNA template. Primers were designed to bind to the coding sequence of target opsins (*RH2A*, *RH2C*, *SWS2B*), and to contain T3 or T7 RNA polymerase promoter sequences at their 5′ ends (T3, reverse primer; T7, forward primer) to allow subsequent strand-specific RNA transcription from cDNA templates for riboprobe synthesis. Anti-sense riboprobes were synthesised and labelled with digoxigenin-UTP (DIG) or fluorescein-UTP (FL) using DIG/FL RNA labelling mix (Sigma-Aldrich). A single *RH2C* riboprobe, targeting both expressed *RH2C-1* and *RH2C-2* paralogues, was synthesised owing to high sequence similarity between these. Hybridised, labelled riboprobes were detected using anti-digoxigenin (Sigma-Aldrich) or anti-fluorescein/Oregon Green (ThermoFisher) antibodies conjugated to horseradish peroxidase. Fluorescent tagging was performed using Alexa Fluor 594 or 488 dyes with tyramide signal amplification (Invitrogen). Finally, retinas were mounted in 70% glycerol in PBS, photoreceptor side up, on microscopy slides with a coverslip.

Dual (*RH2A*/*RH2C*) or single (*SWS2B*) labelled photoreceptor cells were visualised and imaged using a CFI Apo Lambda S LWD 40X/1.15 NA water immersion objective on a spinning disk confocal microscope (Diskovery, Andor Technology, UK) built around a Nikon Ti-E body (Nikon Corporation, Japan) equipped with two Zyla 4.2 sCMOS cameras (Andor Technology), and controlled by Nikon NIS Elements software. Images were exported in TIF file format and further processed (merging of colour channels, adjusting of brightness, *z*-stack projection) with ImageJ v.1.52p (National Institutes of Health, Bethesda, MD, USA).

### Prediction of visual pigment maximal absorbance

Maximal absorbances (λ_max_) of expressed *R. aculeatus* visual pigments were predicted by comparing opsin amino acid sequences with those of percomorph fish species for which the peak spectral sensitivities of their (A1 chromophore-based) visual pigments were known from *in vitro* pigment reconstitution (*Oryzias latipes*, RH1: 502 nm λ_max_, AB180742.1, Matsumoto et al., 2006; *Oreochromis niloticus*, SWS2B: 425 nm λ_max_, JF262088.1, RH2B: 472 nm λ_max_, JF262086.1, RH2Aalpha: 528 nm λ_max_, JF262086.1, LWS: 560 nm λ_max_, JF262088.1, Spady et al., 2006) and by applying tuning effects to their respective peak spectral sensitivities for substitutions at known tuning sites documented in the literature or substitutions at known tuning sites that match the polarity shift of documented substitutions (e.g. for reviews, see Takahashi and Ebrey, 2003; Yokoyama, 2008; Yokoyama and Jia, 2020). For an overview of considered sites and applied effects, see Table S3. We used *O. niloticus* RH2Aalpha rather than RH2Abeta as the reference owing to its greater amino acid sequence similarity to *R. aculeatus* RH2A. We used *O. niloticus* RH2B as the reference for RH2C1 and RH2C2 owing to the lack of *in vitro* characterised RH2Cs (Musilova and Cortesi, 2021 preprint). Opsin gene sequences were translated into amino acid sequences and aligned using MAFFT (v.7.450) (Katoh and Standley, 2013) in Geneious Prime (v.21.1.1). Bovine rhodopsin (NP_001014890.1) was included in all alignments to identify amino acid residues corresponding to known tuning sites and transmembrane regions according to its crystal structure (Palczewski et al., 2000).

### Topographic distribution of ganglion cells and cone photoreceptors

#### Preparation of retinal whole-mounts

Retinal whole-mounts were prepared according to standard protocols (Coimbra et al., 2006; Stone et al., 1981; Ullmann et al., 2012). The orientation of the retina was kept by noting the position of the falciform process inside the eyecup once the cornea and lens were removed. In all our *R. aculeatus* specimens, the falciform process ended in the ventral margin of the retina. Each retina was bleached overnight at room temperature in a solution of 3% hydrogen peroxide in 0.1 mol l^−1^ PBS. For photoreceptor analysis, retinas were whole-mounted (photoreceptor layer up) in 100% glycerol on a microscope slide. For ganglion cell analysis, retinas were whole-mounted, ganglion cell layer facing up, on a gelatinised slide, left to dry overnight in formalin vapour to improve fixation and cell differentiation (Coimbra et al., 2006, 2012), stained in 0.1% Cresyl Violet (Coimbra et al., 2006) and cover slipped with Entellan New (Proscitech). Possible shrinkage during staining was considered negligible and, if present, confined to the retinal margins, as the retinal whole-mount was attached to the slide during the entire staining process (Coimbra et al., 2006).

#### Stereological analyses and topographic map construction

Following the protocols described in de Busserolles et al. (2014a,b), topographic distribution of single cones, double cones, total cones and ganglion cells were assessed using the optical fractionator technique (West, 1991) modified by Coimbra et al. (2009, 2012). Briefly, cone photoreceptors and ganglion cells were randomly and systematically counted using the parameters listed in Table 1 and a 63× oil objective (numerical aperture 1.40) mounted on a compound microscope (Zeiss Imager.Z2) equipped with a motorised stage (MAC 6000 System, Microbrightfield, USA), a digital colour camera (Microbrightfield) and a computer running StereoInvestigator software (Microbrightfield). The counting frame and grid size were chosen carefully to maintain the highest level of sampling and achieve an acceptable Schaeffer coefficient of error (CE<0.1; Glaser and Wilson, 1998). The grid size was adjusted between individuals to take into consideration the variation in size between specimens and allow sampling of around 200 sites per retina (Table 1). To obtain a more accurate estimate of the retinal ganglion cell peak density, sub-sampling was performed in the highest cell density area using the same counting frame but with half the grid size from Table 1.

**Table 1.**
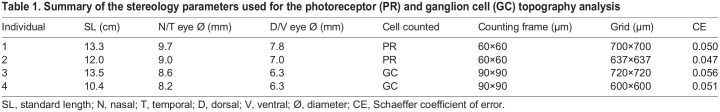
Summary of the stereology parameters used for the photoreceptor (PR) and ganglion cell (GC) topography analysis

Single cones and double cones were easily distinguished and counted separately and simultaneously using two different markers to generate data for single cones alone, double cones alone and the two cell types combined (total cones). Ganglion cells were arranged in a single layer within the ganglion cell layer that also comprised displaced amacrine cells and glial cells. Because amacrine cells were not easily distinguished from ganglion cells using cytological criteria alone (Collin and Collin, 1988; Hughes, 1975), especially in high density areas, they were included in the cell counts and only glial cells were excluded. Although the inclusion of amacrine cells in the analysis usually does not influence the overall topography (Collin and Pettigrew, 1989), it may contribute to a slight overestimation of the peak density of ganglion cells and, ultimately, to a slight overestimation of spatial resolving power.

Topographic maps were constructed in R v.2.15.0 (https://www.r-project.org/) with the results exported from the Stereo Investigator Software according to Garza-Gisholt et al. (2014). The Gaussian kernel smoother from the Spatstat package was used (Baddeley and Turner, 2005) and the sigma value was adjusted to the grid size.

#### Estimation of spatial resolving power

The upper limit of the spatial resolving power (SRP) in cpd was estimated for each individual using the peak density of ganglion cells (PDG in cells mm^−1^) as described by Collin and Pettigrew (1989). Briefly, the angle *a*, subtending 1 mm on the retina can be calculated as follows:
(4)

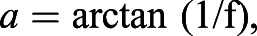
where *f*, the focal length or the distance from the centre of the lens to the retina, is 2.55 (Matthiessen's ratio; Matthiessen, 1882) times the radius of the lens. Knowing *a*, the PDG and the fact that two ganglion cells are needed to distinguish a visual element from its neighbour, the SRP in cpd can be calculated as follows:
(5)

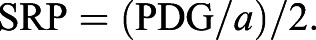


### Behavioural measurements of achromatic and chromatic acuity

#### Experimental setup

We used eight triggerfish (8.1 to 16.5 cm, SL) to measure achromatic and chromatic acuity using a pairwise discrimination behavioural task with square wave gratings (4×4 cm; Fig. S2). Fish were allowed to acclimatise to their tanks for 2–3 weeks and were fed twice a day with a mixture of blended squid, prawns, fish flakes and peas. Tanks were illuminated with 240 V/50 Hz LED Batten 20 W lights (FL2527; Fuzion Lighting, QLD, Australia), which were hung at 50 cm above the end of the tank at which the target stimuli were placed. The side-welling irradiance of each tank was measured with an Ocean Optics USB2000 spectrometer with a 400 µm fibre and a cosine corrector (Fig. S2). Measurements were taken in the middle of the water column at 10 cm from the end of the divider pointing towards the stimuli.

Each tank was divided into three sections: a holding area and two passages separated by a 50-cm-long white opaque Corflute board, down which fish could swim to reach the target stimuli (Fig. S2). During testing, a transparent Perspex board was initially placed across the tank at the end of the Corflute divider, which allowed the fish to swim back and forth in front of both stimuli several times before the transparent Perspex board was lifted and they could swim down one passage, which was considered a choice by the fish. This set the point of decision to 50 cm.

We tested black–white square wave achromatic gratings and two combinations of chromatic square wave gratings: green–yellow to mainly stimulate the medium and long cones, and pink–purple to stimulate the short and long cones. Stimuli were between 0.5 to 5 cpd for black–white achromatic stimuli (*n*=10 stimuli) and 0.5 and 3 cpd for chromatic stimuli increasing in increments of 0.5 cpd (*n*=6 stimuli). A control (‘solid’) stimulus was made using a square wave grating of 11 cpd, which was deemed unresolvable by the triggerfish from previous data (Champ et al., 2014). Overall, the total number of fish that were tested for each colour combination was: *n*=4 for achromatic, *n*=5 for green–yellow and *n*=5 for pink–purple.

Square wave gratings were printed on Steinbeis TrendWhite paper using a Canon LaserJet Pro 400 printer and laminated with 80 μm gloss laminating sheets. We tried to ensure colours within a chromatic combination were as isoluminant as possible (green–yellow and pink–purple) based on quantum catches for an average of double cone members, which are thought to process luminance information (e.g. van den Berg et al., 2020). To calculate quantum catches, the spectral reflectance of printed colours was measured under a black-cloth-covered box with an Ocean Optics USB2000 spectrophotometer (Ocean Optics, FL, USA), a PX-2 pulsed xenon light source and a 200 µm diameter, bifurcated cable held at 45 deg 1 mm above the paper. A Spectralon white standard was used to calibrate the spectrophotometer, and a piece of black velvet covering the end of the fibre was used as the dark standard. Quantum catches of photoreceptors were calculated using photoreceptor spectral sensitivities of triggerfish (Cheney et al., 2013) and illumination and reflectance spectra of printed colours as per eqn 1 in Vorobyev and Osorio (1998). Average normalised quantum catch for the double cone output for the grating colours were: pink 19.3 and purple 18.3, green 27.5 and yellow 27.3, and black 0.8 and white 72.5.

#### Training

In the first round of testing, each fish was randomly assigned to one of three treatments: black–white (*n*=2), green–yellow (*n*=3) or pink–purple (*n*=3) stimuli (fish were retrained to another treatment in a second round of testing). Within each of these groups, one fish was assigned the control (11 cpd) stimulus as the rewarded (S+) stimulus and the remaining individuals were assigned the striped (0.5 cpd) stimulus as the rewarded (S+) stimulus. This was done to account for any bias in the type of stimulus (control/striped) being learnt. Fish were trained using operant conditioning to approach and peck at a stimulus to receive a food reward. Initially, a small piece (<5 mm) of squid mantle was stuck (using the natural adhesive properties of squid) on the rewarded stimulus, while the unrewarded stimulus was left without. Once fish had associated the S+ stimulus with a reward, the squid was removed from the stimulus and fish were then only presented with a reward from above on forceps once they had pecked the S+ stimulus.

Before commencing each trial, an opaque corflute board was placed in front of the transparent Perspex board to ensure the fish could not see the stimuli being placed at the end of each passage. Stimuli were placed in the left or right passage using a random number generator to ensure that fish did not develop a side bias. Once stimuli were in place, the opaque board was lifted, and the fish were given 20 s to view the stimulus through the transparent Perspex board. The transparent board was then lifted and the side that the fish swam down was recorded. Fish did not peck the stimulus in all cases but were given a food reward for approaching the correct stimulus. In instances when a fish swam down one passage, but then changed direction and then went down the other passage during a trial, only the original choice was recorded. If a fish had not swum down any passage within 1 min of the transparent board being lifted, the fish was deemed unmotivated and trials for that fish were stopped for the rest of that session and continued again in the next session. Once fish successfully chose the S+ stimulus >80% of the time over four sessions, fish moved on to the testing phase. It took 3–4 weeks to train most fish (total of 20–29 sessions for each); however, one fish only took 1 week (nine sessions) to reach the required standard for testing.

#### Testing

Testing trials were conducted as per training methods, and one to two sessions were conducted each day with a minimum of 2 h between sessions. The training stimulus (0.5 cpd) was used as reinforcement through the testing phase and was presented 20–98 times per fish, depending on the individual. Each grating between 1.0 and 3.0 cpd was presented to each fish 7–20 times for black–white achromatic stimuli and 9–30 times for chromatic stimuli. Once fish had completed testing with their first treatment stimuli (black–white, green–yellow or pink–purple), they were randomly assigned to one of the other two treatments, then trained and tested again as above. However, two fish only completed one treatment owing to the time taken for training and testing. In total, 2418 trials were conducted with each grating presented 110±14 times (mean±s.e.m.) (Table S4).

### Data analysis

Data analysis for the behavioural experiment was performed in RStudio v.1.3.1056 (https://www.rstudio.com/). We used the R package quickpsy v.0.1.5.1 (Linares and Lopez-Moliner, 2016) to produce logistic functions for all fish for each target colour set (goodness of fit: deviance <8.6, *P*>0.47). Our response variable was whether the fish chose (1) or did not choose (0) the positive S+ stimulus. We then used the function ‘threshold’ to interpolate the 62% threshold, which corresponds to a correct choice frequency significantly different from random behaviour, assuming a binomial distribution of the pooled data per grating (*n*=110, *P*<0.01, one-tailed binomial test). Once thresholds were calculated for each fish, we tested for significant differences between treatments using a general linear mixed model with the lme4 package v.1.1-27.1 (Bates et al., 2015) and the function lmer. Fish ID was included as a random variable. We produced *P*-values using the lmerTest package v.3.1-3 (Kuznetsova et al., 2017). There was no difference in whether fish were trained to receive a food reward from the control stimulus (11 cpd) or the grated stimulus (*t*_4.7_=0.33, *P*=0.76); fish did not perform differently within a colour treatment between the first and second sessions (*t*_3.0_=1.31, *P*=0.28) and there was no effect of the size of the fish on the visual acuity (*t*_4.6_=0.53, *P*=0.62). Therefore, these factors were excluded from the final analysis.

## RESULTS

### Opsin gene repertoire

**Fig. 1.**
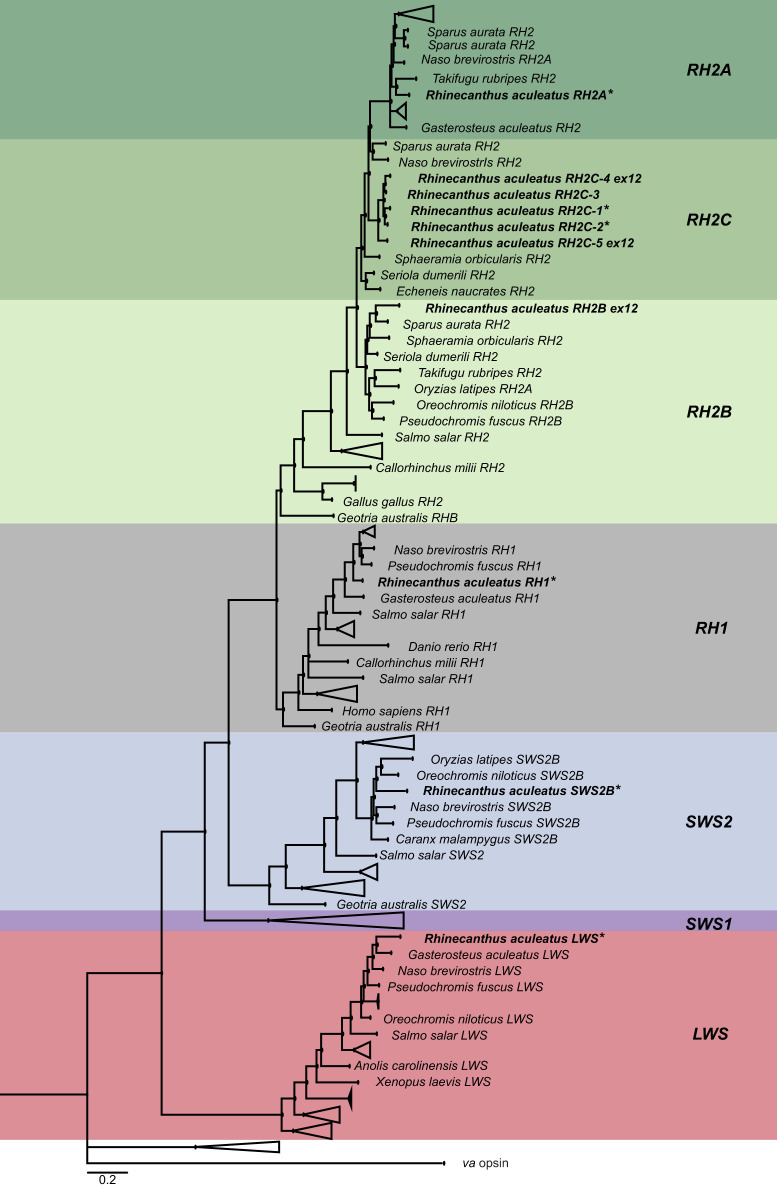
**A phylogenetic tree of the visual opsin genes seen in vertebrates.** The opsin genes belonging to *Rhinecanthus aculeatus* are highlighted in bold. The sequences extracted from the genome and transcriptome (indicated by *) are positioned within their respective opsin class. *RH1*, rhodopsin 1 (rod opsin); *RH2*, rhodopsin-like 2; *SWS2*, short-wavelength sensitive 2; *LWS*, long-wavelength sensitive; *va*, vertebrate ancient opsin (outgroup). The black circles represent the Bayesian posterior probabilities >0.8. Scale bar: 0.2 substitutions per site.

The *R. aculeatus* genome contained one rod opsin gene (*RH1*) and at least nine cone opsins: one violet-sensitive *SWS2B* gene, seven duplicates of the blue-green-sensitive *RH2* gene (*RH2A*, *RH2B* and *RH2C1-5*) and one red-sensitive *LWS* gene*.* For *RH2B* and *RH2C-4* and *RH2C-5*, only the first two exons could be annotated using the whole-genome read-mapping approach. This is because the intron between exons 2 and 3 of *RH2* is too long to be reconstructed using the short reads alone (for details, see Musilova and Cortesi, 2021 preprint). A high-resolution *R. aculeatus* genome based on long-read technology will be needed in the future to resolve these genes and to assess whether even more copies are present. Except for *RH2B* and three *RH2C* copies (*RH2C3-5*), all other opsins were recovered from the transcriptomes. The phylogenetic reconstruction confirmed the class of each opsin gene identified (Fig. 1).

### Opsin gene expression

**Fig. 2.**
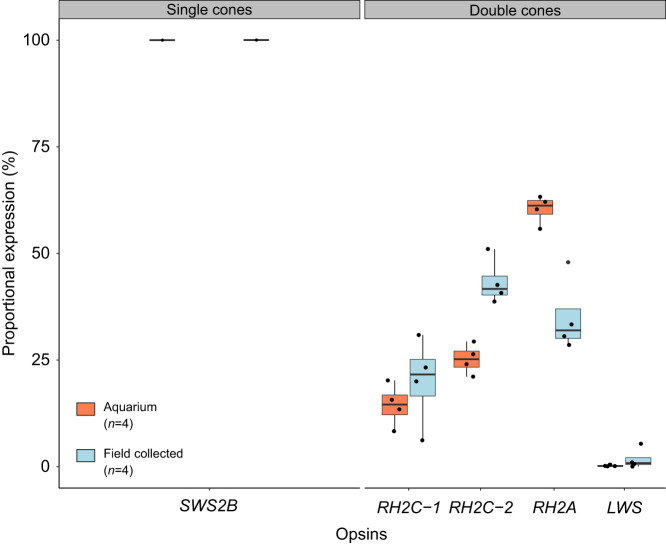
**The proportional cone opsin gene expression of *R. aculeatus* individuals collected from the field and the aquarium.** All individuals expressed one single cone opsin gene, *SWS2B*, and four double cone opsin genes, *RH2C-1*, *RH2C-2*, *RH2A* and *LWS.* The proportional expression of the *RH2* and *LWS* differed between aquarium and field-collected individuals.

The retina of *R. aculeatus* was rod dominated, where *RH1* was expressed on average 68.9±3.75% (*n*=8, mean±s.e.m.). *SWS2B* accounted for 100% of the cone opsin expression in single cones. For the double cone opsins, there was change in expression of the *RH2* and *LWS* genes in relation to whether fish were housed in aquaria or sampled directly from the field. Aquarium fish kept for behavioural trials showed higher *RH2A* expression (*n*=4, 60.30±1.65%) compared with individuals sampled directly from the field (*n*=4, 35.04±4.38%). However, *RH2C-2* was expressed at higher levels for individuals collected directly from the field (45.89±2.12%) compared with aquarium individuals (27.38±3.81%). *RH2C-1* was expressed at similar levels between field site collected (17.34±4.55%) and aquarium individuals (12.14±2.17%). In addition, *LWS* was expressed at low levels for both aquarium and field site collected individuals (field site: 1.70±1.23%, aquarium: 0.18±0.09%) (Fig. 2).

### FISH

**Fig. 3.**
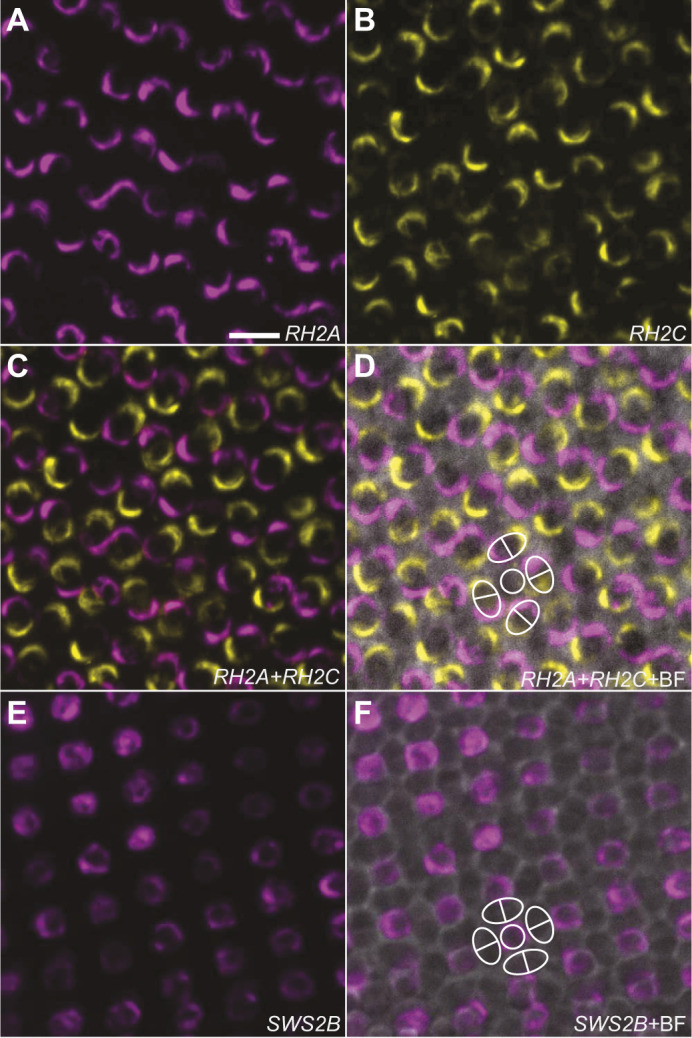
**Double-labelling *in situ* hybridisation of expressed opsin mRNAs in retinal double cone and single cone photoreceptors in *R. aculeatus*.** (A–D) *RH2A* (magenta) and *RH2C* (yellow) mRNA were expressed in opposite members of double cones across the retina. (E,F) *SWS2B* (magenta) mRNA was exclusively expressed in single cone photoreceptors across the retina. Representative single and double cones are outlined with white circles and white ovals, respectively. BF: bright field. Scale bar (A–F): 10 µm.

Double labelling RNA FISH of expressed opsin mRNAs in *R. aculeatus* whole-mount retinas showed that medium wavelength opsins identified from the retinal transcriptome, *RH2A* and *RH2C*s (*RH2C* probes did not discriminate between the two identified *RH2C* paralogues), were expressed in opposite members of double cone photoreceptors across the retina (Fig. 3A–D). The sole single cone opsin identified from the retinal transcriptome, *SWS2B*, was expressed exclusively in single cones (Fig. 3E,F).

### Prediction of visual pigment maximal absorbance

Amino acid sequence similarity between *R. aculeatus* opsins and the used reference opsins ranged from 85.5% (51 variable amino acids) for RH2C-1 and RH2C-2 to 94.1% (21 variable amino acids) for RH1 (Table S3). Among variable amino acids, 33% (RH1) to 47% (LWS) (data not shown) lay outside transmembrane regions and were thus unlikely to impact spectral tuning. The numbers of variable amino acids across transmembrane regions ranged from 15 in RH1 to 32 in SWS2B, the majority of which were substitutions that did not incur a physicochemical change. One (RH1), five (SWS2B), nine (RH2A), 12 (RH2C-1) and 11 (RH2C-2) were at known tuning sites. However, many of these were undocumented substitutions that did not incur a polarity shift, resulting in consideration of one (RH1), four (SWS2B), three (RH2C-1), two (RH2C-2) and two (RH2A) sites for λ_max_ prediction (Table S3). *Rhinecanthus aculeatus* LWS did not differ from the reference LWS at any known tuning sites.

Predicted *R. aculeatus* visual pigment peak sensitivities only in part matched the cone and rod sensitivities obtained via MSP (Cheney et al., 2013). RH1 was predicted to be maximally sensitive at 500 nm owing to S299A (−2 nm; Dungan et al., 2016; Fasick and Robinson, 1998), and closely matched the rod λ_max_ (498 nm) determined using MSP. SWS2B was predicted to be maximally sensitive at 403 nm, primarily tuned to shorter wavelengths compared with *O. niloticus* SWS2B (425 nm) by W265T (−29 nm) and F46V (+8 nm) (Yokoyama et al., 2007). The predicted λ_max_ differed from single cone λ_max_ of 413 nm obtained via MSP. RH2C visual pigments were predicted to be maximally sensitive at 474 nm (RH2C-1) and 476 nm (RH2C-2). Both RH2Cs were slightly red-shifted compared with *O. niloticus* RH2B by primarily M88C (+3 nm; Chinen et al., 2005). RH2C-1 and RH2C-2 differed by only six amino acids (98.3% similarity), with one of these, T266V in RH2C-1, predicted to cause a 2 nm reduction of its λ_max_ (Chinen et al., 2005). The predicted λ_max_ are 6 nm (RH2C-1) and 4 nm (RH2C-2) shorter than the peak sensitivity of the shorter medium-wavelength-sensitive (MWS) double cone determined via MSP (480 nm). RH2A was predicted to be maximally sensitive at 526 nm, with only two sites, C88A (–3 nm; Chinen et al., 2005) and I112V (+1 nm; Chinen et al., 2005), considered to cause a small net blue shift compared with *O. niloticus* RH2Aalpha (528 nm). This predicted λ_max_ almost matched the peak sensitivity of the second, longer MWS double cone in *R. aculeatus* (528 nm) as determined via MSP. As *R. aculeatus* LWS did not differ from *O. niloticus* LWS at any known tuning sites, its predicted λ_max_ was 560 nm.

We identified several substitutions that, although not previously investigated via site-directed mutagenesis (SDM) and *in vitro* pigment regeneration, owing to their incurred polarity shifts and the site of their occurrence, may explain the discrepancies between MSP and predicted λ_max_ (see Table S3). In RH1, S166A incurred a polarity shift at a site close to the known RH1 tuning site A164S (Chan et al., 1992) and is also a substitution known to be involved in RH2 tuning (Yokoyama and Jia, 2020). *Rhinecanthus aculeatus* SWS2B showed three polarity changing substitutions, C163F, S166F and S168A, in immediate proximity to the documented tuning site G164A (Yokoyama and Tada, 2003). In both RH2Cs, V185C caused a polarity shift, whereas previously SDM in zebrafish RH2s had shown a −4 nm blue shift for substitution from T to C at this site (Chinen et al., 2005). C98A incurred a polarity shift at a site adjacent to the powerful RH2 tuning site T97A (−8 nm; Takenaka and Yokoyama, 2007). In *R. aculeatus* RH2A, A151T incurred a polarity shift at a site at which SDM from N to S caused a +4 nm red shift in zebrafish RH2 (Chinen et al., 2005).

### Retinal topography and anatomical spatial resolving power

**Fig. 4.**
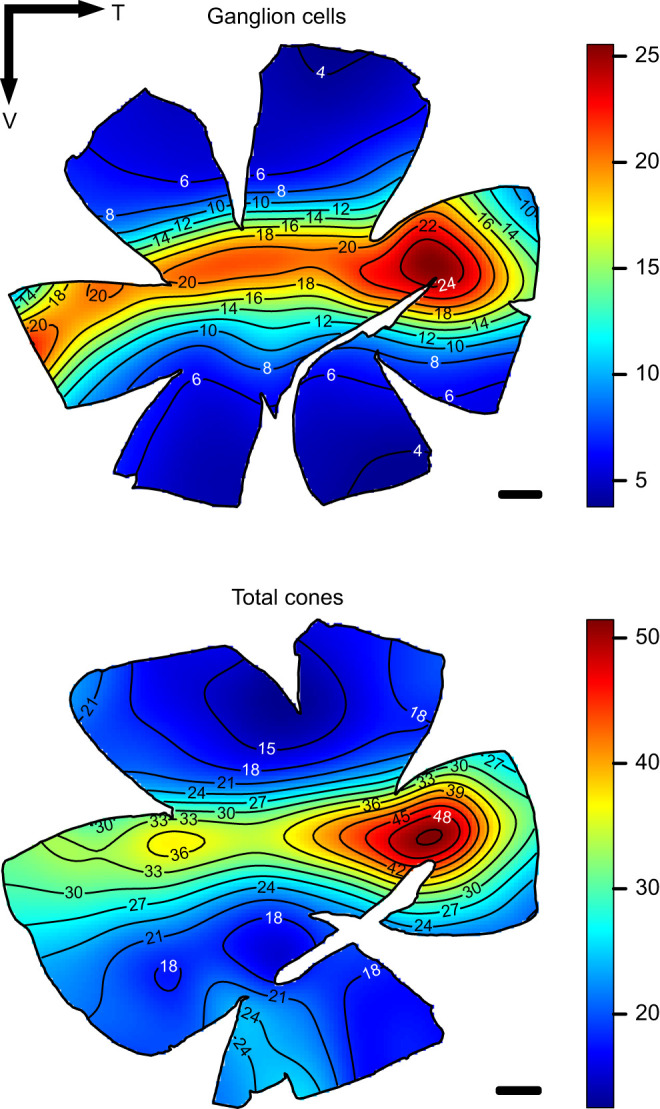
**Topographic distribution of ganglion cells and total cone photoreceptors in *R. aculeatus*.** The black lines represent iso-density contours and values are expressed in densities ×10^3^ cells mm^−2^ (see colour scale). The black arrow indicates the orientation of the retinas. T, temporal; V, ventral. Scale bars: 1 mm.

The topographic distribution of ganglion cells and cone photoreceptors was analysed in four individuals (two individuals per cell type). Because intraspecific variability in topography pattern was very low for both ganglion cells and cone photoreceptors, only one representative map is presented in Fig. 4 and individual maps are provided in Fig. S1.

Ganglion cell topographic distribution revealed the presence of a well-developed horizontal streak with a peak cell density located in the temporal part of the retina (Fig. 4) that ranged from 37,161 to 40,125 cells mm^−2^ in the two individuals analysed (Table 2). Based on these peak cell densities, estimated spatial resolving power for *R. aculeatus* ranged from 6.4 to 7.2 cpd (Table 2).

**Table 2.**
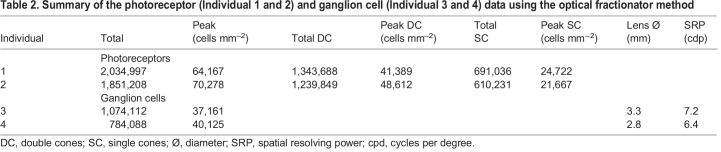
Summary of the photoreceptor (Individual 1 and 2) and ganglion cell (Individual 3 and 4) data using the optical fractionator method

Cone photoreceptors in *R. aculeatus* were arranged in a regular retinal mosaic where one single cone was surrounded by four double cones, resulting in a double cone to single cone ratio of 2:1. This mosaic pattern was consistent across the entire retina, resulting in similar topographic distributions for each cone type. As a result, we only present and describe the distribution pattern for the total cone population (Fig. 4). Maps for each cone type are provided in Fig. S1. The total cone topography varied slightly from the ganglion cell pattern by having a less developed horizontal streak and a more pronounced area temporalis (Fig. 4). However, the peak cell density of total cones, which ranged from 64,167 to 70,278 cells mm^−2^ (Table 2), was found in the same location (i.e. temporal) as the peak density of ganglion cells. Even though ganglion cells and cone photoreceptor topographies were not analysed in the same retina or individuals, comparison of two individuals of similar size (i.e. ∼13 cm SL, individuals 1 and 3) suggested a summation ratio of total cones to ganglion cells in the peak density area of around 2:1.

### Behavioural measurements of achromatic and chromatic acuity

**Fig. 5.**
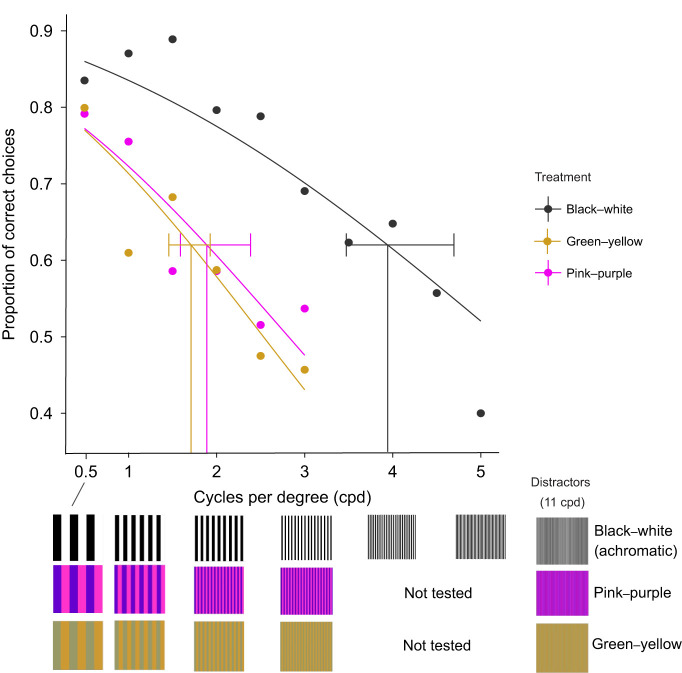
**The probability of success in discriminating between target (S+) gratings and distractor gratings.** A logistic curve was fitted to pooled data across all fish for each colour combination. Behavioural thresholds (62% correct choice) are shown as vertical lines, error bars are 95% confidence intervals. Data for individual fish are shown in Table S4.

Behavioural thresholds (62% correct choice) were significantly higher for black–white achromatic stimuli (3.94 cpd, 95% confidence intervals, CI: 3.47–4.49 cpd) than for green–yellow (1.71 cpd, CI: 1.46–1.93 cpd) (*t*_9.31_=−7.39, *P*<0.001) or for pink–purple stimuli (1.89 cpd, CI: 1.59–2.38 cpd) (*t*_7.51_=−6.00, *P*<0.001). Green–yellow and pink–purple treatments were not significantly different from each other (*t*_6.29_=0.58, *P*=0.58) (Fig. 5).

## DISCUSSION

Previous MSP measurements of *R. aculeatus* photoreceptors suggested three spectrally distinct cone types: one blue-sensitive single cone (λ_max_=413 nm), one blue–green-sensitive double cone member (λ_max_=480 nm) and a second, green-sensitive double cone member (λ_max_=528 nm), as well as a single spectral type of rod photoreceptor (λ_max_=498 nm) (Cheney et al., 2013). Our transcriptome analysis largely – but not fully – reflects this by revealing the expression of a single rod opsin (*RH1*) and for the cone opsins, a short-wavelength-sensitive opsin (*SWS2B*), yet four medium-long-wavelength-sensitive cone opsins (*RH2C-1*, *RH2C-2*, *RH2A* and *LWS*). Except for one field caught individual, *LWS* expression was at levels barely high enough to reconstruct this gene's coding sequence, suggesting that measured double cone λ_max_ were primarily (if not entirely) due to the three identified *RH2* opsins. Amino-acid-based λ_max_ predictions clarified this picture, indicating that the RH2A visual pigment with a λ_max_ of 526 nm was likely to account fully (i.e. not co-expressed) for the green-sensitive double cone member, and this was further supported by our FISH analysis. Predictions also indicated that the two RH2C paralogues were very close in terms of their λ_max_, and as both showed high expression, either one or both may account for the blue–green-sensitive cone member. Unfortunately, owing to high mRNA sequence similarity (97.3% identity) our FISH analysis did not allow a distinction between the two expressed *RH2C* paralogues. Co-expression of either of the two *RH2C* paralogues with *RH2A*, in theory, could have explained the discrepancy between predicted RH2C λ_max_ and measured λ_max_ of the blue–green-sensitive MWS double cone, a pattern seen, for example, in some freshwater cichlids (Dalton et al., 2014; Torres-Dowdall et al., 2017). However, our FISH analysis did not show evidence of *RH2A*/*RH2C* co-expression. Instead, it showed that these opsins were expressed in opposite double cone members, a pattern also observed in other reef fish such as some anemonefishes (Stieb et al., 2019). Taken together, the transcriptome data, visual pigment λ_max_ predictions and FISH analysis corroborate the existing, MSP-based picture of the Picasso triggerfish photoreceptor spectral sensitivity repertoire.

However, although the number of spectrally distinct photoreceptors might be limited, differences in *RH2* expression between individuals that were sampled in the field and the ones that were kept in aquaria shows that opsin gene expression in adult *R. aculeatus* is plastic over time. As for some freshwater fishes (e.g. cichlids; Nandamuri et al., 2017) and reef fishes (e.g. cardinalfishes and damselfishes; Luehrmann et al., 2018), the change in opsin gene expression in *R. aculeatus* was likely caused by differences in the light environment between the reef and the aquarium. The higher expression of *RH2A* in aquarium-held fishes suggests an overall quantitative shift to longer wavelength sensitivity, i.e. more LWS cones when kept under longer-shifted green/red-dominated LED lights. Although it remains to be investigated whether a change in opsin gene expression truly causes a change in visual discrimination, the findings do highlight the importance of assessing an animal's visual system – from molecule to behaviour – in its specific light environment.

### Prediction of visual pigment maximal absorbance

We do note that the reliability of predicted visual pigment λ_max_ based on amino acid sequences is inherently limited despite tuning sites and their effects having been extensively studied in many taxa, and in fish arguably more so than in any other vertebrate group (for reviews, see Takahashi and Ebrey, 2003; Yokoyama, 2000; Yokoyama and Jia, 2020). Omission of yet unknown tuning sites, effects (if any) of unknown substitutions at known tuning sites, and overestimation or underestimation of tuning effects owing to unknown synergistic effects (including effects that affect visual processing in non-spectral ways, e.g. via modulation of retinal release dynamics; Castiglione and Chang, 2018), are among the shortcomings of this approach (e.g. Chinen et al., 2005; Yokoyama and Jia, 2020). For example, for both RH2Cs the substitution of polar C at site 185, where SDM of polar T to polar C has previously shown to shift λ_max_ by as much as 4 nm (Chinen et al., 2005), could explain some or all of the discrepancy between predictions and MSP. Similarly, the polarity-changing substitutions identified in *R. aculeatus* SWS2B could, on their own or by reducing the effect of W265T, cause an increase of the pigment's λ_max_. Consequently, one would consider predictions more robust for more conserved genes (e.g. RH1) or genes in which the extent of λ_max_ variability across many different taxa could be nearly fully described by variations at only a few sites, as is the case for many LWS pigments (‘five-sites rule’; Yokoyama and Radlwimmer, 1998). In contrast, λ_max_ of SWS pigments (SWS1, SWS2As, SWS2B) and RH2 pigments are notoriously difficult to predict owing to a plethora of variable sites that have, to some extent, been implicated with spectral tuning, but complicated by apparent yet not fully understood, synergistic effects (Shi et al., 2001; Yokoyama and Jia, 2020; Yokoyama and Tada, 2003). In the future, additional *in vitro* expression and pigment regeneration assays, particularly in non-model organism species, as well as further investigation of possible tuning sites via SDM, could improve the reliability of spectral sensitivity predictions.

### Retinal topography and anatomical acuity

In this study, ganglion cell topography showed a complete and well-developed horizontal streak and no additional high-density areas outside the streak. The total cone topography had a less developed horizontal streak and a more pronounced area temporalis. The peak ganglion cell density was found in the temporal retina with an average of 38,643 cells mm^−2^, resulting in an acuity estimate of 6.8 cpd. These estimates fit within the typical range of shallow water reef fishes, which have an anatomical estimate of visual acuity between 4 and 27 cpd (Collin and Pettigrew, 1989).

The well-defined streak pattern of *R. aculeatus* found in this study is very similar to the pattern found for another triggerfish, *Balistoides conspicillum* (Collin and Pettigrew, 1989). According to the ‘terrain theory’ (Hughes, 1977), the topography of cells across the retina represents the symmetry of the habitat. Triggerfishes live in open flat areas, where they scour the seafloor for potential prey items and have an uninterrupted view of the sand–water horizon; therefore, a horizontal streak may help them scan their environment for potential predators while searching for prey.

For cone densities and distribution, our results were similar to a previous study on *R. aculeatus* (Champ et al., 2014); however, they substantially differed for the ganglion cell analysis, especially in density and acuity estimates. Ganglion cell topography in Champ et al. (2014) revealed the presence of a partial horizontal streak that extends from the nasal to the central retina, as well as several other high-density areas outside the streak. A peak cell density of 12,450 cells mm^−2^ was found in the temporal retina, resulting in an acuity estimate of 3.4 cpd. These differences in topography pattern, ganglion cell density and acuity estimate between the two studies could be due to several factors such as differences in individual size, the methods of analysis used and/or issues encountered during sample preparation.

In teleost fishes, ganglion cell densities and topography patterns usually show very little intraspecific variability (e.g. de Busserolles et al., 2014b), except between individuals of different life stages and therefore sizes (Shand, 1997; Stieb et al., 2019; Tettamanti et al., 2019). In terms of cell densities, while larger individuals with larger eyes usually have a higher total number of ganglion cells in their retina, the number of ganglion cells per retinal area is usually smaller (Stieb et al., 2019; Tettamanti et al., 2019). According to this, the lower cell densities found in the previous study compared with ours could have been explained if they had used larger individuals. However, Champ et al. (2014) used similar sized individuals or slightly smaller ones compared with our study (7–12 cm versus 10–17 cm SL), indicating that size is not a likely explanation for the differences in densities observed between the two studies.

In both studies, displaced amacrine cells were included into the ganglion cell counts. Contrary to the present study, Champ et al. (2014) subsequently applied a multiplication factor of 0.76 to all their ganglion cell counts to account for the inclusion of displaced amacrine cells, which could explain some of the discrepancies in densities between the two studies. However, even if we take this correction into consideration in our study, our peak ganglion cell density is still at least twice what was reported in the previous study. We chose not to apply a correction factor in this study as displaced amacrine cells in coral reef fishes are mainly present in peripheral and non-specialised areas of the retina, and their inclusion in ganglion cell retinal topographic analyses usually has very little impact on the general topography pattern or the peak cell density estimation (Collin and Pettigrew, 1988). Consequently, even though the inclusion of amacrine cells in our study might slightly overestimate the peak density of ganglion cells and therefore acuity, we believe it provides a more accurate estimate than applying a general correction factor.

The most likely explanation for the differences in ganglion cell densities and topography pattern observed between the two studies is sample processing. During the preparation of our retinal whole-mounts, we found that *R. aculeatus* had a very thick vitreous that proved challenging to remove, especially in the centre of the retina along the retinal meridian. During several attempts which were not included in this study, leftover vitreous resulted in very weak ganglion cell staining making counting unreliable or even unfeasible in certain areas. Similar challenges might have been encountered in Champ et al. (2014), resulting in an under-sampling of the ganglion cell population along the streak. This could explain why an incomplete streak and patchier topography pattern was found in that study, as well as why relatively similar ganglion cell densities (i.e. ∼5000 cells mm^−2^) were found in the dorsal and ventral part of the retina in the two studies, but much lower densities were found along the streak in the previous study.

### Behavioural measurements of achromatic and chromatic acuity

For achromatic gratings, we found that *R. aculeatus* had a behavioural acuity of 3.9 cpd, which was significantly lower than anatomical measurements of 6.8 cpd. For fish, anatomical measurements are often higher than those determined using behavioural experiments (e.g. Brokovich et al., 2010; Pankhurst et al., 1993; Parker et al., 2017). Matthiessen's ratio (2.55), which is an average value from a measured range of 2.4–2.82, is used to estimate focal length instead of using the true focal length of the fish lens, and therefore may cause discrepancies in estimations. Visual acuity estimates based on ganglion cell counts only represent a theoretical upper limit of visual acuity. This is because not all ganglion cells contribute to visual acuity. For example, in primates, spatial resolving power is set by the midget ganglion cells, which only account for 70–80% of the total ganglion cell population (Wässle, 2004). Furthermore, the function of different types of retinal ganglion cells varies both temporally and spatially across the retina in zebrafish (Zhou et al., 2020).

Behavioural studies of visual acuity are likely to represent a more accurate estimate of an animal's functional visual abilities than its anatomical visual acuity. Furthermore, experiments that utilise ecologically relevant paradigms to measure acuity should give closer estimates between behavioural and anatomical measured thresholds. For example, Temple et al. (2013) found that the maximum acuity of archerfish was similar to anatomical measurements when fish spat at prey using the area centralis in the ventro-temporal region of the retina. In contrast, optomotor/optokinetic tests provide some measure of average retinal acuity but will not capture the acuity in specialised regions of the retina, such as in the area centralis or fovea. Behavioural measurements of acuity may also vary depending on the stimuli used. Triggerfish did not perform as well in acuity tests when trained to circular stimuli as opposed to grating stimuli of horizontal and vertical stripes (Champ et al., 2014). Furthermore, different acuities were measured when honeybees were presented with radial (sectored) compared with linear (square-wave) gratings (Srinivasan and Lehrer, 1988), which could be explained by orientation-specific feature detecting receptive fields (Marr and Hildreth, 1980).

We found that achromatic acuity (3.94 cpd) of triggerfish was significantly higher than chromatic acuity for both the green–yellow (1.71 cpd) and pink–purple (1.89 cpd) stimuli. This supports previous findings in birds, mammals and bees (Giurfa et al., 1997; Lind and Kelber, 2011; Mullen, 1985); however, in humans and birds, differences in acuity were found between different colour channels. In budgerigars, visual acuity was lower for blue–green than for red–green gratings, which may be explained by lower numbers of SWS cones in the retina compared with MWS and LWS cones (Hart et al., 2000). Similarly in humans, acuity was lower for blue–yellow than for red–green contrasts (Mullen, 1985). However, the black–white achromatic stimulus had higher overall luminance compared with the chromatic stimuli based on double cone quantum catch; this may have also improved acuity for achromatic stimuli.

In conclusion, we hope that data from this study will inform future studies with this species investigating a range of topics including, but not limited to, teleost perception, navigation and cognition. We believe that Picasso triggerfish will continue to play a key role in contemporary research in these areas owing to their versatility as a focal study organism. We highlight the need for further information on the specific chromatic opponent mechanisms in animals, which is currently poorly understood (Baden and Osorio, 2019).

## Supplementary Material

Supplementary information
