# Supplementary material for: Seeing Picasso: an investigation into the visual system of the triggerfish Rhinecanthus aculeatus
Source: J Exp Biol. 2022 Apr 8;225(7):jeb243907. doi: 10.1242/jeb.243907 (PMC9080752; doi:10.1242/jeb.243907)
Supplement: Supplementary information [file jexbio-225-243907-s1.pdf]

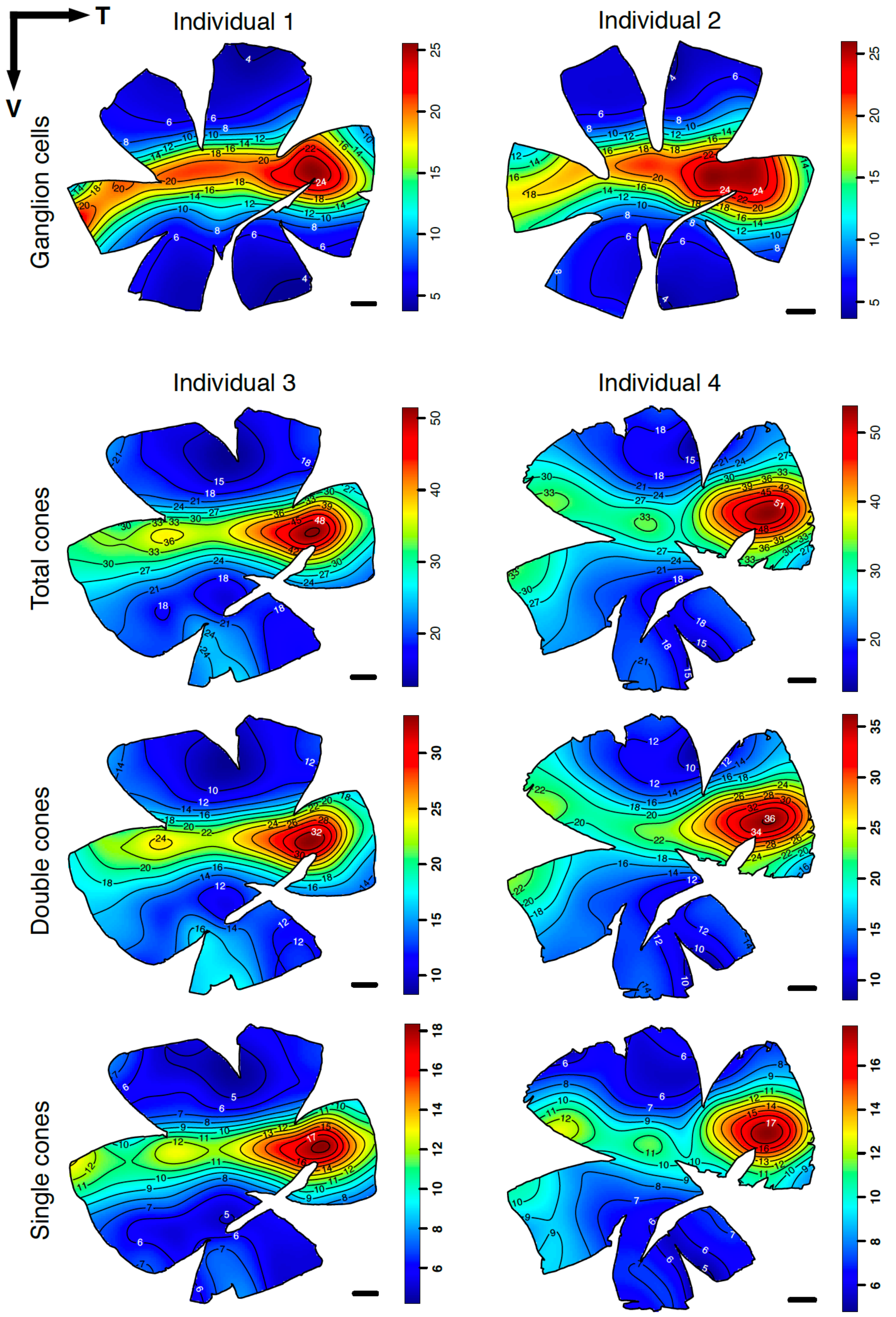

Fig. S1. Intraspecific variability topographic distribution of ganglion cells and cone photoreceptors (single, double and total cones) in *Rhinecanthus aculeatus*. The black lines represent iso-density contours and values are expressed in densities x 10^3 cells/mm^2. The black arrow indicates the orientation of the retinas. T = temporal, V = ventral. Scale bars: 1 mm.

Journal of Experimental Biology • Supplementary information

Behavioural measurements of achromatic and chromatic acuity

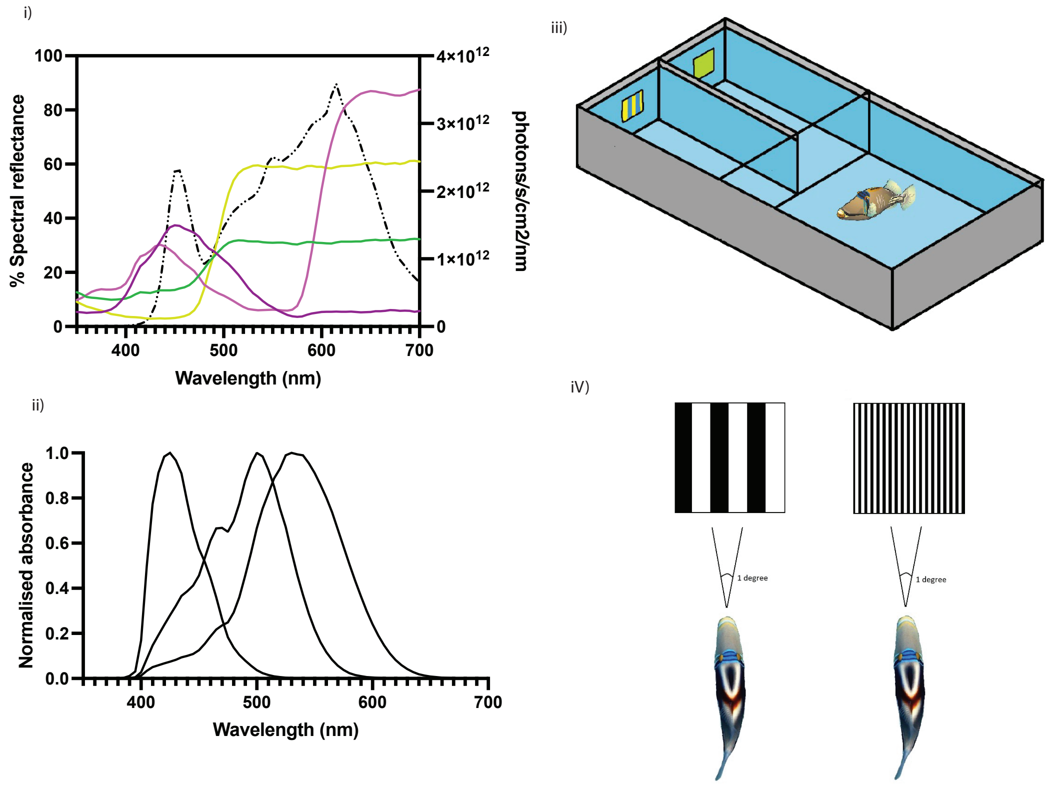

Fig. S2. i) Spectral reflectance of laminated stimuli used in each treatment labelled by color: pink, purple, green and yellow; irradiance of sidewelling lights measured in the testing tanks shown as dashed line; ii) Spectral sensitivities of the three cones in triggerfish, *Rhinecanthus aculeatus*, with a single cone sensitivity of λ_{max} = 413 nm, and double cone sensitivities of λ_{max} = 480 nm and λ = 528$_{max}$ nm multiplied by yellow cornea (Cheney et al., 2013); iii) Experimental tank setup; iv) An example of the achromatic (black and white) square-wave gratings: a) 1 cycle per degree; b) 5 cycles per degree.

Journal of Experimental Biology • Supplementary information

Table S1. Primers used for probe template synthesis of fluorescence in-situ hybridisation. T7 (forward primer) and T3 (reverse primer) RNA polymerase promoter sequences are shown in bold.

Target gene	Primer	Sequence
RH2A	RH2A_F1<br>RH2A_R1	5'-**TAATACGACTCACTATAGGG**ATGTACAAGCTCCTGGCTTG-3'<br>5'-**AATTAACCCTCACTAAAGGG**TCTTTGCAAAGAAGGCGCAC-3'
RH2C-1 & RH2C-2	RH2C_F1<br>RH2C_R1	5'-**TAATACGACTCACTATAGGG**CCATCAACTTCCTGACGCTA-3'<br>5'-**AATTAACCCTCACTAAAGGGAGCCAAACACCATCAGGACA**-3'
SWS2B	SWS2B_F1<br>SWS2B_R1	5'-**TAATACGACTCACTATAGGG**AGAGGCCCCAGATGACTTCT-3'<br>5'-**AATTAACCCTCACTAAAGGG**ACGGACTGACTCGATGAGGA-3'

Journal of Experimental Biology • Supplementary information

Table S2. Summary of transcriptomes, opsin mapping and opsin gene expression of *Rhinecanthus aculeatus*.

			**Mapping**						**Proportional opsin expression % (normalized to coding sequence length)**						
			**Rods**	**Single cones (SC)**	**Double cones (DC)**				**Rod vs Cone**		**SC**	**DC**			
**Origin**	**ID**	**# filtered transcriptome reads**	***RH1* # reads**	***SWS2B* # reads**	***RH2A* # reads**	***RH2C-1* # reads**	***RH2C-1* # reads**	***LWS* # reads**	**R**	**C**	***SWS2B***	***RH2A***	***RH2C-1***	***RH2C-2***	***LWS***
Field	FZ9	10736408	14826	560	1488	1037	1901	44	74.62	25.38	100	33.29	15.65	50.08	0.97
	FZ10	9975040	23638	1390	1876	1894	2376	0	75.77	24.23	100	30.52	26.33	43.15	0.00
	FZ11	6864530	15196	798	654	458	1170	14	83.04	16.96	100	28.49	22.01	48.90	0.60
	1LIT	22709442	136187	18897	26723	3429	22709	3014	79.07	20.93	100	47.86	5.38	41.44	5.32
	Mean	**12571355**	**47696.50**	**5411.25**	**7685.25**	**1704.50**	**8751.67**	**768.00**	**78.13**	**21.87**	**100**	**35.04**	**17.34**	**45.89**	**1.72**
	Se	**3481577.01**	**29879.20**	**4498.64**	**6351.03**	**646.09**	**5229.22**	**748.72**	**1.89**	**1.89**	**-**	**4.39**	**4.55**	**2.12**	**1.22**
Aquarium	LY	21584148	273003	40177	82429	18303	35983	52	60.61	39.39	100	60.27	11.87	27.83	0.04
	SQ	21581834	272743	34687	79679	28871	34329	212	60.47	39.53	100	55.69	6.19	37.97	0.15
	PE	24905208	305271	40809	81017	20063	26989	124	64.30	35.70	100	63.20	14.43	22.28	0.10
	NE	20168834	387049	66207	168363	22443	79481	1170	53.34	46.66	100	62.03	16.11	21.44	0.43
	Mean	**22060006**	**309516.50**	**45470.00**	**102872**	**22420**	**44195.50**	**389.50**	**59.68**	**40.32**	**100**	**60.30**	**17.34**	**45.89**	**0.18**
	S.e.	**1005269.25**	**26948.78**	**7047.68**	**21837.55**	**2311.58**	**11923.10**	**262.22**	**2.29**	**2.29**	**-**	**1.65**	**2.17**	**3.81**	**0.09**

Table S3. Predicted *R. aculeatus* visual pigment peak spectral sensitivities (λ_{max}) compared to reference visual pigments (*O. latipes* RH1; *Oreochromis niloticus,* SWS2B, RH2B, RH2Aalpha, LWS), *R. aculeatus* λ_{max} determined via MSP, and tuning sites and effects considered for predictions. [1] (Matsumoto et al., 2006), [2](Spady et al., 2006), [3](Dungan et al., 2016), [4](Fasick and Robinson, 1998), [5](Yokoyama et al., 2007), [6](Yokoyama and Tada, 2003), [7](Chinen et al., 2005), [8](Yokoyama and Jia, 2020), [9](Cheney et al., 2013)

	RH1	SWS2B	RH2C-1	RH2C-2	RH2A	LWS
Similarity to reference amino acid sequence (%)	94.1	86.1	85.5	85.5	91.5	89.9
Total variable amino acid	21	49	51	51	30	36
Variable amino acid in transmembrane regions	15	32	30	29	18	19
Variable amino acids at known tuning sites	1	5	12	11	8	0
Reference pigment peak absorbance (nm λ_{max})	502[1]	425[2]	472[2]		528[2]	560[2]
Known tuning sites and applied tuning effects (nm)	S299A (-2)[3,4]	F46V (+8)[5]<br>A109G (-2)[5]<br>G164A (+1)[6]<br>W265T (-29)[5]	M88C (+3)[7]<br>I112V (+1)[7]<br>T266V (-2)[7]	M88C (+3)[7]<br>I112V (+1)[7]	C88A (-3)[7]<br>I112V (+1)[7]	-
Candidate tuning sites – no effects documented	S166A	C163F<br>S166F<br>S168A	C98A<br>V185C	C98A<br>V185C	A151T	-
Predicted peak absorbance (nm λ_{max})	500	403	474	476	526	560
MSP peak absorbance (nm λ_{max})	498[9]	413[9]	480[9]		528[9]	-

Table S4. Summary of stimuli presented to each fish and in which order, whether the fish was trained to receive a food reward from the Distractor (11 cpd) or the test gratings (0.5-6 cpd), the total number of trials conducted by each fish for each colour (total number of trials = 2438) and calculated discrimination thresholds at 62% correct choice. NA indicates not tested due to time taken to complete treatment 1.

Fish ID	Size (SL, cm)	Treatment 1	S +ve	Threshold (cpd)	Treatment 2	S +ve	Threshold (cpd)
Billy	16	Green-yellow (179)	Control (11 cpd)	2.19	NA	NA	NA
Bitey	10	Pink-purple (120)	Grating	2.45	Green-yellow (167)	Grating	2.17
Diego	16	Achromatic (203)	Grating	4.89	Pink-purple (75)	Grating	2.73
Ernie	17	Achromatic (205)	Control (11 cpd)	5.30	NA	NA	NA
Gilbert	16.5	Green-yellow (184)	Grating	2.71	Achromatic (235)	Grating	5.03
Lyra	10	Pink-purple (120)	Grating	2.95	Green-yellow (167)	Grating	3.04
Mike	15	Green-yellow (188)	Grating	2.58	Pink-purple (167)	Grating	3.27
Sophie	15	Pink-purple (193)	Control (11 cpd)	5.44	Achromatic (235)	Control (11 cpd)	5.44

Journal of Experimental Biology • Supplementary information

Further references

Cheney, K. L., Newport, C., McClure, E. C. and Marshall, N. J. (2013). Colour vision and response bias in a coral reef fish. *J Exp Biol* **216**, 2967-2973.

Chinen, A., Matsumoto, Y. and Kawamura, S. (2005). Reconstitution of ancestral green visual pigments of zebrafish and molecular mechanism of their spectral differentiation. *Mol Biol Evol* **22**, 1001-10.

Dungan, S. Z., Kosyakov, A. and Chang, B. S. W. (2016). Spectral tuning of killer whale (orcinus orca) rhodopsin: Evidence for positive selection and functional adaptation in a cetacean visual pigment. *Mol Biol Evol* **33**, 323-336.

Fasick, J. I. and Robinson, P. R. (1998). Mechanism of spectral tuning in the dolphin visual pigments. *Biochemistry* **37**, 433-438.

Matsumoto, Y., Fukamachi, S., Mitani, H. and Kawamura, S. (2006). Functional characterization of visual opsin repertoire in medaka (oryzias latipes). *Gene* **371**, 268-278.

Spady, T. C., Parry, J. W., Robinson, P. R., Hunt, D. M., Bowmaker, J. K. and Carleton, K. L. (2006). Evolution of the cichlid visual palette through ontogenetic subfunctionalization of the opsin gene arrays. *Mol Biol Evol* **23**, 1538-47.

Yokoyama, S. and Jia, H. (2020). Origin and adaptation of green-sensitive (rh2) pigments in vertebrates. *FEBS Open Bio* **10**, 873-882.

Yokoyama, S. and Tada, T. (2003). The spectral tuning in the short wavelength-sensitive type 2 pigments. *Gene* **306**.

Yokoyama, S., Takenaka, N. and Blow, N. (2007). A novel spectral tuning in the short wavelength-sensitive (sws1 and sws2) pigments of bluefin killifish (lucania goodei). *Gene* **396**, 196-202.

Journal of Experimental Biology • Supplementary information
